# 

**Published:** 1868-08

**Authors:** 


					﻿VOL. XXII.
No. 8.
THE
DENTAL REGISTER
EDITED BY
J. TAFT & G. WATT.
AUGUST, 1868.
MONTHLY:
THREE DOLLARS PER ANNUM, IN ADVANCE.
CINCINNATI:
WRIGHTSON & co., Printers, 167 WALNUT STREET.
«T. A TFJlf. TAFT, jOsntist.s, 33S IFest Fourth St.
				

